# Hyper-reactive Malarial Splenomegaly (HMS) in a patient with β thalassaemia syndrome

**DOI:** 10.11604/pamj.2014.19.310.5576

**Published:** 2014-11-21

**Authors:** Yaw Ampem Amoako, George Bedu-Addo

**Affiliations:** 1 Department of Medicine, Komfo Anokye Teaching Hospital, Kumasi, Ghana; 2 School of Medical Sciences, Kwame Nkrumah University of Science and Technology, Kumasi, Ghana

**Keywords:** Hyper-reactive malarial splenomegaly, béta thalassemia syndrome, Proguanil, Massive splenomegaly, Ghana

## Abstract

This report describes a case of hyper-reactive malarial splenomegaly in a patient with a thalassaemia syndrome. Increased haemoglobin A_2_ is valuable for the diagnosis of common forms of β-thalassemia, while haemoglobin F (HbF) helps in diagnosis of the rarer δβ- forms. Thalassemia is characterised by splenomegaly and is common in malaria endemic areas. Hyper-reactive malarial splenomegaly is also a common cause of massive splenomegaly in malaria endemic areas. Splenic enlargement regresses with prolonged antimalarial therapy

## Introduction

The oxygen carrying capability of the red blood cells (RBCs) relies on haemoglobin, a tetramer protein that comprises 4 globin chains bound to the haem molecule. There are 4 major types of globins: alpha (α), beta (β), gamma (γ), and delta (δ). The dominant haemoglobin in adults (haemoglobin A) is composed of 2 alpha and 2 beta chains. Two minor forms of haemoglobin constitute a small percentage of normal blood: haemoglobin F (fetal), composed of 2 alpha chains and 2 gamma chains, and haemoglobin A2, composed of 2 alpha chains and 2 delta chains. A very tightly controlled globin chain production process keeps the ratio of alpha chains to non-alpha chains at 1.00 (± 0.05). Thalassemia, by altering this process, disrupts this ratio. Decreased production of alpha globin gene products yields a relative excess of beta chains, which results in less stable chains; this leads to the clinical disease known as alpha thalassemia [1]. Similarly, impaired production of beta globin gene products manifests with a more severe disease known as beta thalassemia [2, 3]. Individuals with thalassemia syndrome are most often of African, Asian, Mediterranean, or Middle Eastern descent. Beta thalassaemia trait results from reduced or absent function of one of the two beta globin genes. The individual is heterozygous for a beta thalassaemia gene, β^0^ or β +. Heterozygotes have no or only mild clinical problems and are often diagnosed only on the basis of their red cell indices and other biochemical tests. The mean cell volume (MCV) and mean corpuscular haemoglobin (MCH) are reduced and the red blood cell count is increased. Increased haemoglobin A_2_ is valuable for the diagnosis of common forms of β-thalassemia, while haemoglobin F (HbF) helps to diagnose the rarer δβ- forms. HMS is a common cause of massive splenomegaly in malaria endemic areas. The underlying defect appears to be a lack of T suppressor cells involved in controlling antibody production by B lymphocytes [1]. Splenic enlargement regresses with prolonged antimalarial therapy [2, 3, 4]. In this paper we describe the case of a young female who was referred to the haematology clinic at Komfo Anokye Teaching Hospital, to be investigated for anaemia and massive splenomegaly.

## Patient and observation

A case of massive splenomegaly with anaemia referred for investigations at the haematology clinic at Komfo Anokye Teaching Hospital is described. HE is a 15-year-old female who presented with a mass in the left hypochondrial region of six months duration. The mass was gradually growing in size. There was no associated fever, night sweats, recurrent respiratory tract infections or weight loss. Her past medical history was unremarkable. She had her menarche at 13 years and menstrual loss is over a period of 4 days. She denied menorrhagia. She was not on any regular medication. There was no past history of blood transfusion. On examination, she looked well. She had mild pallor but was anicteric. There was no lymphadenopathy. She had splenomegaly palpable 10 cm below the left costal margin. There was no other palpable abdominal mass. There was no ascites. She had a soft ejection systolic murmur at the mitral area. No other abnormalities were detected on examination. Table 1 details the results of laboratory investigations performed. HE had microcytic hypochromic anaemia with a high RDW. She also had a mild thrombocytopenia with normal leucocyte count and a raised reticulocyte count. The iron studies were however normal. Quantification of haemoglobin fractions was by High Performance Liquid Chromatography (HPLC) using a Bio- Rad variant II system. We were unable to perform tests to determine the alpha-beta chain synthesis ratio or do genetic tests of the beta-globin cluster (using Southern blot or PCR assay tests) due to technical and logistic challenges. The possible diagnoses for HE include - β^0^ thalassaemia/hereditary persistence of haemoglobin F or δβ^0^ thalassemia/hereditary persistence of haemoglobin F - HMS She was treated presumptively for HMS with Proguanil 200 mg daily. She was reviewed monthly and she showed consistent reduction in spleen size until the ninth month of therapy when the spleen was impalpable. Her haemoglobin improved slightly to 10.0g/dl and her platelet count increased to 210 x 109/l after 9 months of Proguanil and Folate therapy. Folate supplementation was necessary because of the inhibition of endogenous Folate synthesis by Proguanil. The good response to Proguanil confirmed a diagnosis of HMS and the results of the haemoglobin electrophoresis and quantification point to a diagnosis of β thalassemia.

**Table 1:** Laboratory investigations and results

Parameter	Patient's results	Normal range
Haemoglobin (g/dl)	9.3	13.5- 15.0
Haematocrit (%)	27.6	41- 50
Mean cell volume (fl)	64.5	80- 99
Mean cell haemoglobin concentration (g/dl)	31.5	31.6- 34.9
Red blood cell count (×10^12^/l)	4.57	3.88- 4.99
Red cell distribution width (RDW)	28.6	9.5- 15.5
Platelets (×10^9^/l)	101	144- 400
White cell count (×10^9^/l)	4.2	4- 10
Reticulocyte count (%)	3.8	0.5- 2.5
Total Bilirubin (mol/l)	18	up to 17mol/l
**Haemoglobin electrophoreisis**		
Cellulose acetate membrane (pH 8.2)	FA_2_	
Agar gel (pH 6.0)	F	
Haemoglobin quantification (by HPLC)		
HbA_2_	4.6%	2.2- 3.5%
HbF	90.1%	< 1%
**Iron studies**		
Ferritin	138	15-300 g/l
Transferrin	2	2- 4g/l
Serum iron	14.6	10-30 g/l
Transferrin saturation	28.1	15-30%
IgM level (g/L)	2.3	Local mean is 3.5g/l (for HMS range is 0.61-65.2)

## Discussion

Thalassemia is the most common inherited cause of microcytosis and iron deficiency anemia (IDA) is the most common acquired form of microcytosis. Lifelong microcytosis is suggestive of, a congenital disorder and a diagnosis of thalassemia should be considered [5]. A normal to high RBC and a disproportionately low MCV is useful in making a distinction from iron deficiency anaemia since a high RBC is quite uncommon in IDA [6]. β thalassaemia is caused by any one of more than 200 point mutations and rarely by deletions [7]. β thalassaemia occurs as a trait or symptomatic disease. In β thalassaemia trait, the level of Hb A_2_ may increase from normal value of 2% to a value of 3%-6%. In βthalassaemia disease, the hemoglobin electrophoresis reveals mostly HbF. A slight or moderate increase in hemoglobin F may also be seen in the β thalassaemia trait and in compound heterozygotes. Heterozygotes for β thalassaemia have mild hypochromic anemia with microcytosis and a raised concentration of HbA_2_. HE's predominant haemoglobin is HbF and HbA_2_. HbF is the main haemoglobin in babies and in normal people is replaced by adult haemoglobin HbA. She is not making HbA. Increased HbA_2_ is valuable for the diagnosis of the common forms of thalassemia whilst HbF along with a low MCH brings out the rarer δβ forms. The normal iron studies make iron deficiency anaemia an unlikely cause of her microcytosis. HE has one of the β^0^ thalassemia syndromes. Most patients with β^0^ thalassaemia have severe anaemia requiring transfusions. HE has never been transfused, implying she has a mild form of β^0^ thalassaemia syndrome. Certain genetic conditions such as haemoglobinopathies, including thalassaemia, provide protection against malaria. A case controlled study in Liberia showed that for β thalassaemia, heterozygotes appeared to be relatively protected from both mild and severe malaria [8]. HMS is thought to result from repeated attacks of malaria that lead ultimately to massive splenomegaly of 10cm or more below the costal margin. There is no diagnostic test for HMS. There is usually more than a 40% reduction in spleen size when patients are put on malaria prophylaxis such as Proguanil for at least 6 months [9]. The complete regression of the spleen when HE was treated with Proguanil confirmed the diagnosis of HMS.

## Conclusion

HMS is a common cause of massive splenomegaly in malaria endemic areas whilst theβthalassaemias pose important public health problems because they are common and usually produce severe anaemia in the homozygous and compound heterozygous states. Difficult cases do occur and one needs specialized investigations such as DNA analysis to establish the correct diagnosis.
